# Multiple Arginine Residues Are Methylated in *Drosophila* Mre11 and Required for Survival Following Ionizing Radiation

**DOI:** 10.1534/g3.118.200298

**Published:** 2018-04-25

**Authors:** Qing Yuan, Ran Tian, Haiying Zhao, Lijuan Li, Xiaolin Bi

**Affiliations:** * Department of Biological Sciences, and; † Institute of Cancer Stem Cell, Cancer Center, Dalian Medical University, Dalian 116044, Liaoning, China; ‡ Department of Chemistry and Chemical Engineering, Beijing University of Technology, Beijing 100124, China

**Keywords:** Mre11, arginine methylation, DART1, Glycine-Arginine-Rich motif, ionizing radiation

## Abstract

Mre11 is a key player for DNA double strand break repair. Previous studies have shown that mammalian Mre11 is methylated at multiple arginines in its C-terminal Glycine-Arginine-Rich motif (GAR) by protein arginine methyltransferase PRMT1. Here, we found that the *Drosophila* Mre11 is methylated at arginines 559, 563, 565, and 569 in the GAR motif by DART1, the *Drosophila* homolog of PRMT1. Mre11 interacts with DART1 in S2 cells, and this interaction does not require the GAR motif. Arginines methylated Mre11 localizes exclusively in the nucleus as soluble nuclear protein or chromatin-binding protein. To study the *in vivo* functions of methylation, we generated the single Arg-Ala and all Arginines mutated flies. We found these mutants were sensitive to ionizing radiation. Furthermore, Arg-Ala mutated flies had no irradiation induced G2/M checkpoint defect in wing disc and eye disc. Thus, we provided evidence that arginines in *Drosophila* Mre11 are methylated by DART1 methytransferase and flies loss of arginine methylation are sensitive to irradiation.

Mre11 plays a pivotal role at DNA double strand break repair (haber 1998). It forms a complex with Rad50 and Nbs1 (MRN), and is highly conserved in eukaryotes. The MRN complex executes the sensor function to recognize DNA double strand breaks (DSBs) (petrini and stracker 2003), and regulates DNA damage repair through both homologous recombination and non-homologous end joining pathways (haber 1998). It is widely recognized that post-translational modifications, such as phosphorylation, acetylation, methylation etc, are critical for protein functions (Blanc and Richard 2017). This is also true to Mre11, previous studies have shown that multiple arginines in the C-terminal Glycine-Arginine-Rich motif (GAR) of Mre11 are methylated by protein arginine methyltransferase 1 (PRMT1) in HeLa cells (boisvert *et al.* 2005a; boisvert *et al.* 2005b; Dery *et al.* 2008).

Arginine methylation is catalyzed by the nine-member protein arginine methyltransferase (PRMT) family in mammals (Blanc and Richard 2017). Based on their substrate specificity, PRMTs are classified into three types. Type I and type II PRMTs catalyze the formation of ω-N^G^-monomethylarginine (MMA), which is the intermediate for production of the ω-N′^G^, N^G^-asymmetric dimethylarginine (aDMA) and ω-N^G^, N^G^-symmetric dimethylarginine (sDMA). Type III PRMT catalyzes the formation of MMA only on histones. Among PRMTs, PRMT1 is the major arginine methyltransferase and contributes to more than 90% of type I PRMT activity in human cells (tang *et al.* 2000). The arginines methylated by PRMT1 are usually surrounded by glycines, they together form a GAR motif, which is also called the Arginine- and Glycine-Rich motif (RGG) (thandapani *et al.* 2013). Study in mouse embryonic fibroblasts (MEFs) has shown that loss of PRMT1 causes genome instability, and delayed cell cycle progression (yu *et al.* 2009). PRMT1 performs its functions in response to DNA damage through methylation of DNA repair factors, such as Mre11 (boisvert *et al.* 2005a; Dery *et al.* 2008), p53BP1 (boisvert *et al.* 2005c), p53 (scoumanne and chen 2008), DNA polymerase beta (pol β) (el-andaloussi *et al.* 2007; el-andaloussi *et al.* 2006), HMGA1 (zou *et al.* 2007) etc.

Study in mammalian cells has shown that Mre11 interacts with PRMT1, and mutation of arginines in the GAR motif causes intra-S-phase checkpoint defect and loss of exonuclease activity (boisvert *et al.* 2005a). Although arginine methylation is not required for MRN complex formation, it is essential for its binding with chromatin (boisvert *et al.* 2005a; Dery *et al.* 2008; yu *et al.* 2012). Inhibition of arginine methylation suppresses Mre11 localization to DNA damage sites (8), and reduces γ-H2AX foci formation (boisvert *et al.* 2005b) as well as Rad51 foci formation upon DNA damage (Dery *et al.* 2008; yu *et al.* 2012). Studies in *mre11^RK/RK^* knock-in mice and MEFs have shown that arginines substituted with lysines causes hypersensitivity to gamma ray radiation, and MEF cells display significant increase of aberrant chromosomes, defects in G2/M cell cycle checkpoint and ATR/CHK1 signaling activation, and reduced RPA and RAD51 foci after irradiation (yu *et al.* 2012).

The *Drosophila* arginine methyltransferase (DART) is identified by sequence homology with mammalian protein arginine methyltransferase (boulanger *et al.* 2004). DART1 is the *Drosophila* homolog of PRMT1, it can methylate mammalian PRMT substrates and arginine rich *Drosophila* substrates *in vitro* (boulanger *et al.* 2004). The *Drosophila* Mre11 is highly conserved, we and others have shown that the major defects in *mre11* mutated flies are telomere-telomere associations, aneuploidy (bi *et al.* 2004; ciapponi *et al.* 2004) and G2/M checkpoint defect induced by low dose irradiation (bi *et al.* 2005). While mammalian PRMT1 interacts with Mre11 and catalyzes its methylation, whether highly conserved *Drosophila* Mre11 is methylated remains unknown.

In this study, we identified four arginine residues methylated by DART1 in the GAR motif of *Drosophila* Mre11, and knock-down of *dart1* reduced the level of arginine methylation *in vitro*. Methylated Mre11 localizes exclusively in the nucleus as chromatin-binding protein and nucleoplasmic protein. Moreover, *Drosophila* Mre11 interacts with DART1 in S2 cells, and this interaction does not require the GAR motif. To study the *in vivo* functions of Mre11 arginine methylation, we generated knock-in flies of Arg-Ala (RA) point mutations and 4 Arg-Ala (4RA) mutation by ends-in approach (rong *et al.* 2002; rong and golic 2000). The *mre11^RA^* and *mre11^4RA^* flies are sensitive to ionizing radiation. However, the *mre11^RA^* and *mre11^4RA^* flies do not show DNA damage induced G2/M checkpoint defect in the wing disc and eye disc.

## Materials and Methods

### Plasmids construction

#### Constructs for in vitro methylation assay:

The full length cDNA of *dart1* was PCR amplified with primers dart1-F and dart1-R (Table S1), and cloned into *Xho*I/*EcoR*I sites of pRSETb vector (Invitrogen, Grand Island, NY, USA). The full length cDNA of *mre11* was amplified with mre11-F and mre11-R, *mre11*N206 was amplified with mre11-N-F and mre11-N-R, *mre11*M167 was amplified with mre11-M-F and mre11-M-R, and *mre11*C247 was amplified with mre11-C-F and mre11-C-R (Table S1). *Mre11* was cloned into *EcoR*I/*Sal*I sites, and *mre11*C247 was cloned into *BamH*I/*EcoR*I sites of pSJ8 vector (pET21a derivative, a gift from Dr. Yuhui Dong). *Mre11*N206 and *mre11*M167 were cloned into *Kpn*I/*EcoR*I sites of pRSETb vector.

#### Constructs for co-immunoprecipitation:

The full length cDNA of *dart1* was PCR amplified with primers V5-dart1-F and V5-dart1-R, and cloned into *EcoR*V/*Not*I sites of pAc5.1/V5-His B vector (Invitrogen, Grand Island, NY, USA). *Mre11* was amplified with Myc-mre11-F1 and Myc-mre11-R, and cloned into *EcoR*I/*Not*I sites of pCMV-Myc vector (Clontech, Mountain View, CA, USA) to generate pCMV-Myc-mre11. Then, the correct pCMV-Myc-mre11 plasmid was used as a template and amplified with Myc-mre11-F2 and Myc-mre11-R, and cloned into *EcoR*V/*Not*I sites of pAc5.1/V5-His B vector (Invitrogen, Grand Island, NY, USA) to generate pAc5.1-Myc-mre11. *Mre11*GARΔ was constructed by two-step PCR. In the first PCR, pAc5.1-Myc-mre11 was used as template, primer pairs Myc-mre11-F2 and mre11GARΔ-overlapPCR-R, mre11-GARΔ-overlapPCR-F and Myc-mre11-R were used separately to generate two fragments. In the second PCR, the products from first PCR were mixed together and used as template, primer pair was Myc-mre11-F2 and Myc-mre11-R (Table S1) and the PCR product was cloned into *EcoR*V/*Not*I sites of pAc5.1/V5-His B vector.

### Protein expression and purification

DART1, Mre11, Mre11N206, Mre11M167 and Mre11C247 were expressed in BL21. DART1, Mre11N206 and Mre11M167 protein were purified with HIS-Select Nickel Magnetic Agarose Beads (Sigma, St. Louis, MO, USA), Mre11 and Mre11C247 protein were purified with Amylose Resin (NEB, Ipswich, MA, USA) following the manufacture′s instructions.

### Methylated arginine antibody production

Antibodies recognizing single methylated arginine at Mre11-R559, R563, R565, R569, R571 or R579 sites were generated by GL Biochem (Shanghai, China). The antigenicity of *Drosophila* Mre11 was analyzed using DNAStar software, and two peptides with better epitope segments were selected, their sequences were 555-PATGR*GAAR*GR*GT-567, and 568-AR*TR*AGATAATR*GKG-582 (methylated arginines were labeled with *). Peptides with single aDMA modification at specific arginine site were synthesized and used to immunize rabbits. Methylated arginine antibodies (M-Ab) were precleared with non-arginine methylation peptide and affinity purified with a site specific arginine methylated peptide. Specificity was verified by a dot-blot assay. And non-methylated antibodies (NM-Ab1 and NM-Ab2) were generated with two non-arginine methylation peptides respectively.

### In vitro methylation assay

*In vitro* methylation assay was performed as described (boisvert *et al.* 2005a). Purified Mre11, Mre11N206, Mre11M167 and Mre11C247 were incubated with His-DART1 separately, in 50 mM Tris-HCl pH 8.5, with 0.1 µM of [methyl-^3^H] S-adenosyl methionine (Sigma, St. Louis, MO, USA), to a final volume of 30 μl for 1 h at 30°. The reaction was stopped by adding 2 × SDS-PAGE sample buffer and boiled for 10 min. Samples were subjected to electrophoresis and visualized by fluorography.

### Generation of mre11 mutants

A 6-kb genomic fragment of *mre11* with an I-SceI cut site constructed into pCR2.1 (Gao *et al.* 2009) was used as a template. The R559A, R563A, R565A, R569A, or 4RA change was made by site directed mutagenesis using primers: mre11 R559A-F and mre11 R559A-R, mre11 R563A-F and mre11 R563A-R, mre11 R565A-F and mre11 R565A-R, mre11 R569A-F and mre11 R569A-R, mre11 4RA-F and mre11 4RA-R, respectfully (Table S1). The full length of genomic constructs was sequenced to verify the specified RA change as the only mutation. The 6-kb *Not*I fragment was excised and constructed into the targeting vector pTV2. Targeting crosses and the subsequent reduction crosses were performed as previously described (Gao *et al.* 2009; Gong *et al.* 2005).

The overall integrity of the *mre11* genomic region from homozygotes of intermediate flies and mutants was assayed by Southern blot analysis (Gong *et al.* 2005), using genomic DNA digested with *Bgl*II and probe amplified from *mre11* gene region with primers Probe-F and Probe-R (Table S1). The mutants were further verified by cDNA sequencing and genomic DNA sequencing.

### Fly genetics

All flies were maintained at 25° on standard corn meal unless specified. The fly lines used in this study are: *w^1118^*; *mre11^*Δ*35K1^/TSTL Tb* (Gong *et al.* 2005); *mre11R559^A1-1T10^*, *mre11R563^A3-1D5^*, *mre11R565^A2-7M5^*, *mre11R569^A6-34H1^*, and *mre114RA^2-3P3^* flies were generated in this study, kept as homozygote and used in the assay.

### Embryo fractionation

Embryos of *w^1118^* wild-type flies were collected at 0 ∼4 h and fractionated following the manufacture′s instructions (Thermo Scientific). Briefly, embryos were dechorionated in 3% NaOCl for 2 min, washed thoroughly with tap water, then grounded gently in 300 μL pre-cooled 1 × phosphate-buffered saline (PBS) with a dounce homogenizer, centrifuged at 6,000 g for 5 min at 4°, and supernatant was carefully removed. The pellet was resuspended with 200 μL of CERI and put on ice for 10 min, 11 μL of CERII was added and treated for 1 min to lysate cell membrane. The sample was centrifuged with maximum speed (∼16,000 × g) at 4° for 5 min, and supernatant (cytoplasm) was immediately transfered to a clean pre-chilled tube. The pellet was washed in l mL precooled 1 × PBS three times, resuspended with 200 μL ice-cold NER to lysate nuclei, then vortexed 15 sec in every 10 min for 4 times. The sample was centrifuged at ∼16,000 × g for 10 min, and supernatant (nucleoplasm) was transfered to a clean pre-chilled tube. The pellet was resuspended in 200 μL ice-cold 1 × PBS and sonicated to get the chromatin binding protein.

### RNA interference

S2 cells were seeded into 25 cm^2^ flasks at 1×10^6^ cells/mL the day before experiments. Five million cells were transfected with *dart1* siRNA, 5′-GCAGCGAGGAUACAUACAATT-3′, at final concentration of 40 pmol/L using Lipofectamine RNAiMAX Reagent (Invitrogen, Grand Island, NY, USA). The scrambled sequence of siRNA was used as a negative control (NC). Forty eight hours after transfection, medium was changed and let cells recover for 24 h.

### Reverse transcription PCR

Total RNA was prepared from S2 cells using an RNeasy Plus Mini Kit (Qiagen, Hilden, NW, Germany), and reverse transcribed with Oligo(dT)_15_ primer and M-MLV reverse transcriptase following the manufacturer’s instructions (Promega, Fitchburg, WI, USA). The PCR primers used were dart1-RT-F and dart1-RT-R to detect transcripts of *dart1*, and gapdh1-RT-F and gapdh1-RT-R to detect *gapdh1* as internal control (Table S1). The PCR products were visualized by 2% agarose gel electrophoresis.

### Western blot

Protein samples were subjected to SDS-PAGE electrophoresis and transferred to PVDF membrane. Samples were detected with primary antibodies against Mre11 (1:6,000, rabbit), β-actin (1:10,000, abcam ab8224, Cambridge, MA, USA), Histone H3 (1:15,000, abcam ab1791, Cambridge, MA, USA), MeMre11Arg559 (1:1,000), MeArg563 (1:800), MeArg565 (1:1,000), MeArg569 (1:1,500), MeArg571 (1:1,500), MeArg579 (1:1,500). Secondary antibodies were HRP-conjugated goat anti-rabbit (1:5,000, Jackson, West Grove, PA, USA) and goat anti-mouse (1:10,000, MultiSciences Biotech, Hangzhou, China).

### Co-immunoprecipitation

DART1-V5 and Myc-Mre11 or Myc-Mre11GARΔ constructs were co-transfected into S2 cells with Effectene (Qiagen, Hilden, Germany) for 24 h at 25°. Cells were lyzed in IP lysis buffer (20 mM Tris-HCl, pH 7.5, 150 mM NaCl, 1% Triton X-100), then pre-cleared with Protein-G agarose beads (Roche, Indianapolis, IN, USA) and incubated with V5 antibody (abcam, Cambridge, MA, USA) or Myc antibody (CST, Beverly, MA, USA) for 3 h at 4°. Protein-G dynabeads (Invitrogen, Grand Island, NY, USA) were added and incubated overnight at 4°. They were then washed 4 times with ELB buffer (50 mM HEPES, pH 7.3, 250 mM NaCl, 0.1% NP40).

### Viability assay

Actively crawling third instar larvae of *w^1118^*, *mre11R559A^1-1T10^*, *mre11R563A^3-1D5^*, *mre11R565A^2-7M5^*, *mre11R569A^6-34H1^*, *mre114RA^2-3P3^* were collected, and irradiated at a rate of 1.3 Gy/min with dosage of 0, 10, 20, 30, 40 Gy in a X-Ray Biological Irradiator (X-RAD 320iX, Precision X-ray Inc), respectively. Let the irradiated flies grow to adult on fly medium, the number of viable flies was counted and survival percentage was calculated as viable flies divided by larvae irradiated. At least 100 larvae were treated at each dosage for each genotype, and experiments were repeated three times.

### G2/M checkpoint assay

The G2/M checkpoint assay was performed as described (bi *et al.* 2005). Briefly, third instar larvae were irradiated with a dosage of 10 Gy. Let the irradiated larvae recovered at 25° for 1.5 h. Wing discs and eye discs were dissected in 1 × phosphate-buffered saline (PBS), fixed in 1 × PBS containing 4% formaldehyde at room temperature (RT) for 25 min. Samples were washed three times with 1 × PBS, blocked with 0.3% PBST containing 0.5% Tween-20, 1% BSA, 2% sheep serum, then stained with primary antibody of rabbit anti-histone H3pS10 (1:500, Millipore, 06-570) overnight at 4°. The secondary antibody used was anti-rabbit IgG (1:400, Cell Singnaling Technology, 4412). At least 6 wing discs or eye discs were examined for each genotype and experiments were repeated three times. The samples were analyzed on a Leica SPE5 confocal laser-scanning microscope.

### Quantification by Image J software

The intensity of signals on the film was quantified using Image J software (National Institutes of Health, USA). Protein bands were measured with Uncalibrated OD mode, and the percentage of each fraction is the gray value of each band divided by total value. The confocal images of G2/M cell cycle checkpoint were qualified using Multi-point tool to count the positive cells.

### Statistics

All statistical comparisons were performed using SPSS. P-values were calculated by two-tailed student′s *t*-tests. Data were presented as: *, *P* ≤ 0.05, **, *P* ≤ 0.01, ***, *P* ≤ 0.0001.

### Data availability

Strains and plasmids are available upon request. All supplementary materials necessary for confirming the conclusions have been uploaded to figshare and the DOI is 10.6084/m9.figshare.6157718. Figure S1 contains the SDS-PAGE of purified proteins of Mre11, Mre11N206, Mre11M167 and Mre11C247 with Coomassie staining. Figure S2 contains the dot-blotting detection of methylation antibodies. Figure S3 contains methylated arginines detection of Mre11 in S2 cells. Figure S4 shows the ends-in scheme that was used to generate mutant flies. Figure S5 contains the southern-blot detection to verify the intermediate flies and knock-in alleles. Figure S6 contains G2/M cell cycle checkpoint detection after ionizing radiation in eye disc. Sequences of primers used in this study are listed in Table S1. File S1 contains detailed descriptions of all supplemental files. Supplemental material available at Figshare: https://doi.org/10.6084/m9.figshare.6157718.

## Results

### Drosophila Mre11 is methylated by DART1

**Figure 1:**
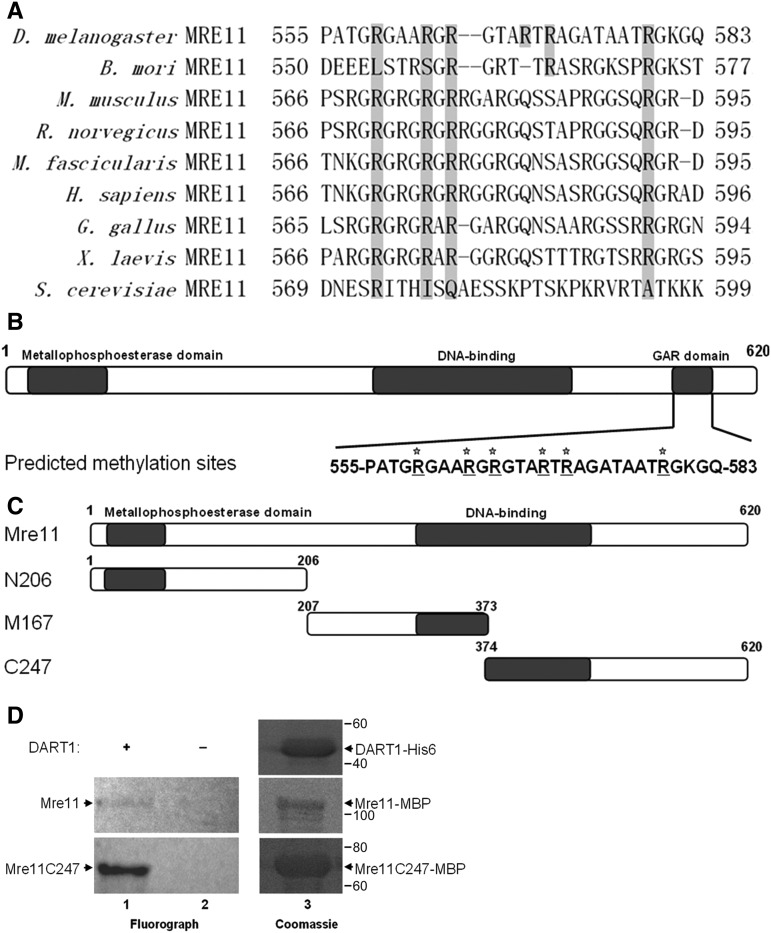
*Drosophila* Mre11 is methylated by DART1. A. Sequence alignment of Mre11 from D. melanogaster, B. mori, M. musculus, R. norvegicus, M. fascicularis, H. sapiens, G. gallus, *X. laevis* and S. cerevisiae. Drosophila Mre11 has a Glycine-Arginine-Rich(GAR)motif at its C-terminus. B. Predicted arginine methylation in the GAR motif. There are six potential methylated arginines in the GAR motif. C. Diagram of construction of Mre11 and truncated Mre11 proteins. Mre11 protein is expressed as full length, N-terminal Aa 206 (N206), middle part (M167), and C-terminal Aa 247 (C247). D. *In vitro* methylation assay. Purified full length Mre11 and truncated Mre11 proteins were incubated with ^3^H-labeled S-adenosylmethionine (SAM) in the presence or absence of DART1. Methylated proteins were subjected to SDS-PAGE and detected by autoradiography. One representative result is shown here, at least three independent experiments were performed. 1. Fluorograph of Mre11 and Mre11C247 methylated by DART1. 2. Fluorograph of Mre11 and Mre11C247 un-methylated without DART1. 3. Coomassie staining of purified DART1, Mre11 and Mre11C247.

The functions of Mre11 are highly conserved in *Drosophila* (bi *et al.* 2005; bi *et al.* 2004; ciapponi *et al.* 2004). To investigate whether highly conserved *Drosophila* Mre11 has a GAR motif and arginines in this motif are methylated, we aligned protein sequence of *Drosophila* Mre11 with that of *B. mori*, *M. musculus*, *R. norvegicus*, *M. fascicularis*, *H. sapiens*, *G. gallus*, *X. laevis* and *S. cerevisiae* using Clustal W2 software (http://www.ebi.ac.uk/Tools/msa/clustalw2/). The *Drosophila* Mre11 has a GAR motif at its C-terminus between Aa 555-583 (Figure 1A), and there are six arginines in the GAR motif. The following analysis using the MEMO software (http://www.bioinfo.tsinghua.edu.cn/∼tigerchen/memo.html) (chen *et al.* 2006) suggested that all six arginines are potential methylation sites of protein arginine methyltransferase (Figure 1B).

To prove that *Drosophila* Mre11 is methylated by DART1, the *Drosophila* homolog of PRMT1, full length of DART1, and full length, N-terminus (N206), central region (M167), C-terminus (C247) of Mre11 were expressed in *E.coli* and purified (Figure 1C and Figure S1). We found that full length Mre11 protein and Mre11C247 protein were methylated by DART1 using *in vitro* methylation assay (Figure 1D), whereas Mre11N206 and Mre11M167 were not (data not shown), indicating that Mre11 is methylated at its C-terminus by DART1, probably in the GAR motif.

### Endogenous Mre11 is methylated at multiple arginines in the GAR motif

To localize the methylated arginines, peptides with single aDMA modified arginine at the Aa 559, 563, 565, 569, 571 or 579 sites were prepared and used separately to generate specific antibodies. All antibodies were incubated with peptide without modification, then affinity purified with specific arginine methylated peptide, and the specificity was verified with a dot-blot assay (Figure S2). Previous studies have shown that methylation is essential for Mre11 binding to chromatin (boisvert *et al.* 2005b; Dery *et al.* 2008), suggesting that methylated *Drosophila* Mre11 might localize specifically in the nucleus and perform its functions.

**Figure 2:**
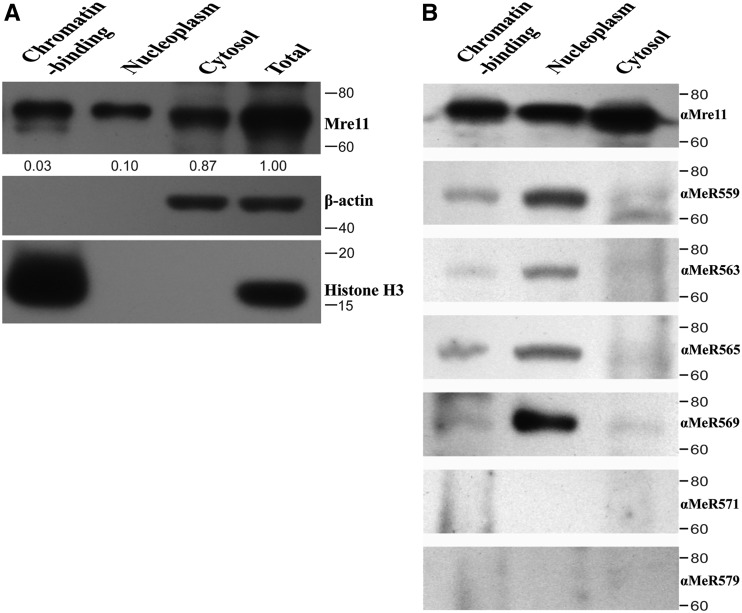
Endogenous Mre11 is methylated at multiple arginines in the GAR motif. A. Subcellular localization of Mre11. Embryos of *w^1118^* wild-type flies were fractionated and detected with anti-Mre11 antibody. Mre11 protein is not equally localized in cytoplasm, nucleocytoplasm and chromatin-binding fraction. To visualize Mre11 protein, nucleocytoplasmic fraction and chromatin-binding fraction were concentrated 2.fivefold and 6.25-fold, respectively, and quantified with Image J software. One representative result is shown here, at least three independent experiments were performed. B. Endogenous Mre11 has multiple methylated arginines recognized by specific arginine methylated antibodies, which are at Aa 559, 563, 565 and 569.

The *Drosophila* embryos of *w^1118^* flies were fractionated into chromatin-binding protein, soluble nucleoplasmic protein and cytoplasmic protein. We found that Mre11 protein was not equally allocated into each fraction, 87% of Mre11 protein is cytoplasmic, 10% is soluble nucleoplasmic, and only 3% of Mre11 protein binds to chromatin, respectively (Figure 2A).

To investigate the methylation status of Mre11, we adjusted each fraction loaded on the gel to get as close amount of Mre11 protein in each fraction as possible. Arginine methylated antibodies specifically recognize R559, R563, R565 and R569 sites of Mre11, whereas antibodies of the other two arginines at R571 and R579 could not detect Mre11 protein (Figure 2B). Moreover, only soluble nucleoplasmic and chromatin-binding Mre11 protein can be detected by arginine methylated antibodies, indicating that methylated Mre11 localizes exclusively in the nucleus as the chromatin-binding protein and soluble nucleoplasmic protein. These four methylated arginine sites were also detected using the S2 cells and their nuclear localization was confirmed (Figure S3).

### Knock-Down of dart1 reduces level of arginine methylated Mre11 in S2 cells

**Figure 3:**
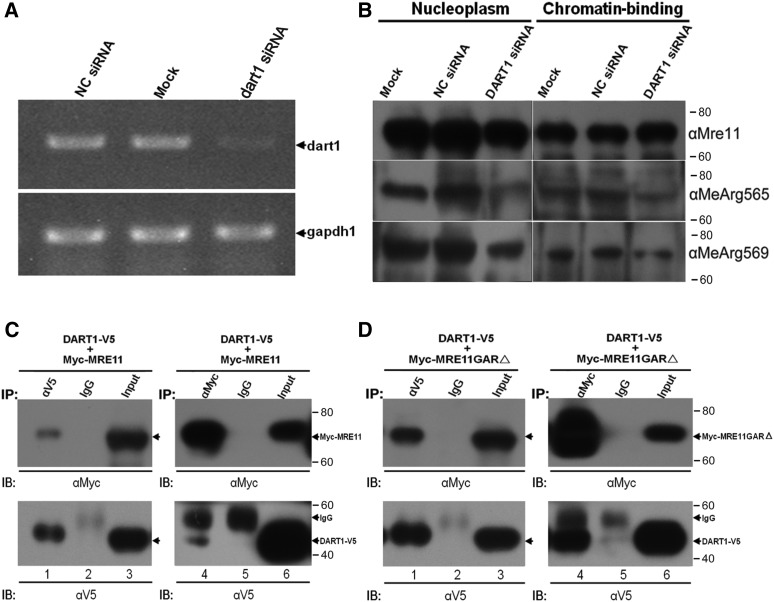
Mre11 interacts with DART1 in S2 cells which does not depend on the GAR motif. A. RT-PCR detection of *dart1* expression after knock-down by siRNA in S2 cells, *gapdh1* was used as the internal control. B. Arginine methylation of Mre11 was reduced when *dart1* was knock-down. Arginine methylation of Mre11 in S2 cells was detected with arginine methylation antibodies specific for Arg565 (αMeArg565) and Arg569 (αMeArg569). C. Co-immunoprecipitation was performed by DART1-V5 and MRE11-Myc in S2 cells. Mre11 protein and DART1 protein were detected by Myc antibody and V5 antibody, respectively. D. Mre11GARΔ protein interacts with DART1 shown by co-immunoprecipitation.

As DART1 catalyzes arginine methylation of *Drosophila* Mre11 *in vitro*, we wish to know whether knock-down of *dart1* expression influences the methylation of arginines of Mre11. The S2 cells were treated with *dart1* siRNA or mock treated for 48 h (Figure 3A), let the cells recover without siRNA for 24 h, and then detected with R565 and R569 specific methylation antibodies. We found that knock-down of *dart1* reduced level of methylated Mre11 in S2 cells (Figure 3B).

### Mre11 interacts with DART1 in S2 cells which does not require the GAR motif

Mammalian Mre11 is methylated by PRMT1, and they interact with each other (boisvert *et al.* 2005a; Dery *et al.* 2008). Our data indicated that *Drosophila* Mre11 is methylated by DART1 (Figure 1D), and knock-down of *dart1* decreased methylated Mre11. We further investigated whether they interact with each other by performing the co-immunoprecipitation assay. Mre11 and DART1 were co-expressed in *Drosophila* S2 cells as Myc-Mre11 and DART1-V5 fusion proteins. Using anti-V5 antibody or anti-Myc antibody, we detected Mre11 protein or DART1 protein, respectively, whereas Mre11 protein or DART1 protein was not pulled-down by a control IgG (Figure 3C), suggesting that the *Drosophila* Mre11 physically interacts with DART1 in S2 cells.

We further investigated whether the GAR motif of the *Drosophila* Mre11 is required for its interaction with DART1. The GAR motif was removed from Mre11 (Mre11GARΔ) by mutagenesis. The Mre11GARΔ and DART1 were co-expressed in *Drosophila* S2 cells as Myc-Mre11GARΔ and DART1-V5 fusion proteins. Using anti-V5 antibody or anti-Myc antibody, we can still detect Mre11GARΔ protein or DART1 protein (Figure 3D). Mre11 lacking a GAR motif retains the ability to interact with DART1, indicating that the GAR motif is not required for Mre11-DART1 interaction in S2 cells.

### Arginine mutated flies are sensitive to ionizing radiation

**Figure 4:**
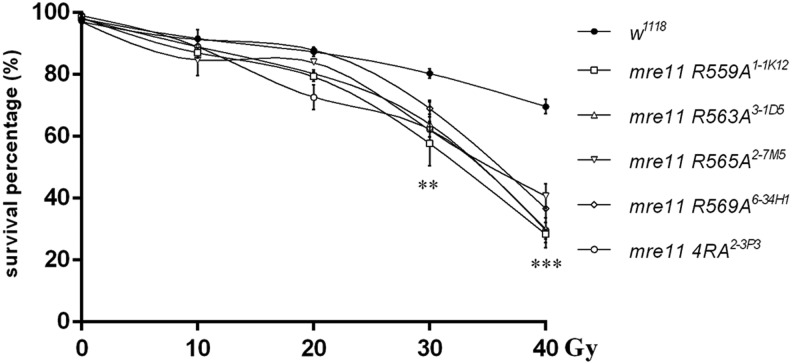
Flies with *mre11* Arg-Ala mutation are sensitive to ionizing radiation. Late third instar larvae of *w^1118^*, *mre11R559A^1-1T10^*, *mre11R563A^3-1D5^*, *mre11R565A^2-7M5^*, *mre11R569A^6-34H1^*, and *mre114RA^2-3P3^* were irradiated with dosage of 0, 10, 20, 30, and 40 Gy, respectively. Survival adults were counted one week after irradiation, and survival percentage was calculated as number of viable adult flies divided by number of irradiated larvae. At least 100 larvae were treated at each dosage for each genotype, and three independent experiments were performed. ** *P* ≤ 0.01, *** *P* ≤ 0.001; error bars indicate SEM.

To explore the *in vivo* functions of arginine methylation of Mre11 in *Drosophila*, we generated knock-in flies of Arg-Ala (RA) point mutation and 4 Arg-Ala (4RA) mutation of the four methylated arginines, by using ends-in strategy as previously described (Figure S4) (rong *et al.* 2002; rong and golic 2000). The single RA and 4RA mutated *mre11* flies were verified by southern-blot, cDNA sequencing and genomic DNA sequencing (Figure S5 and data not shown). All of *mre11^RA^* and *mre11^4RA^* flies were viable and fertile, indicating the arginine methylation of Mre11 is not essential for fly survival. We collected the late third instar larvae of homozygote of *mre11^RA^* and *mre11^4RA^* flies, treated with ionizing radiation at dosage of 0, 10, 20, 30, and 40 Gy, respectively. Let the irradiated larvae grow, counted the survival adults, and calculated the survival percentage. All *mre11^RA^* and *mre11^4RA^* flies were sensitive to irradiation, however they showed different sensitivity. They were sensitive to 30 and 40 Gy (*P* ≤ 0.05), while *mre11^R563A^* flies were also sensitive to 20 Gy (*P* = 0.003), and *mre11^R559A^* and *mre11^4RA^* flies were sensitive to 10 Gy (Figure 4, P ≤ 0.05). Moreover, we did not observe the additive effect in *mre11^4RA^* mutant, suggesting that methylated arginines play the redundant role at DNA damage response.

### Arginine methylation is not required for DNA damage induced G2/M cell cycle checkpoint in wing disc and eye disc

**Figure 5:**
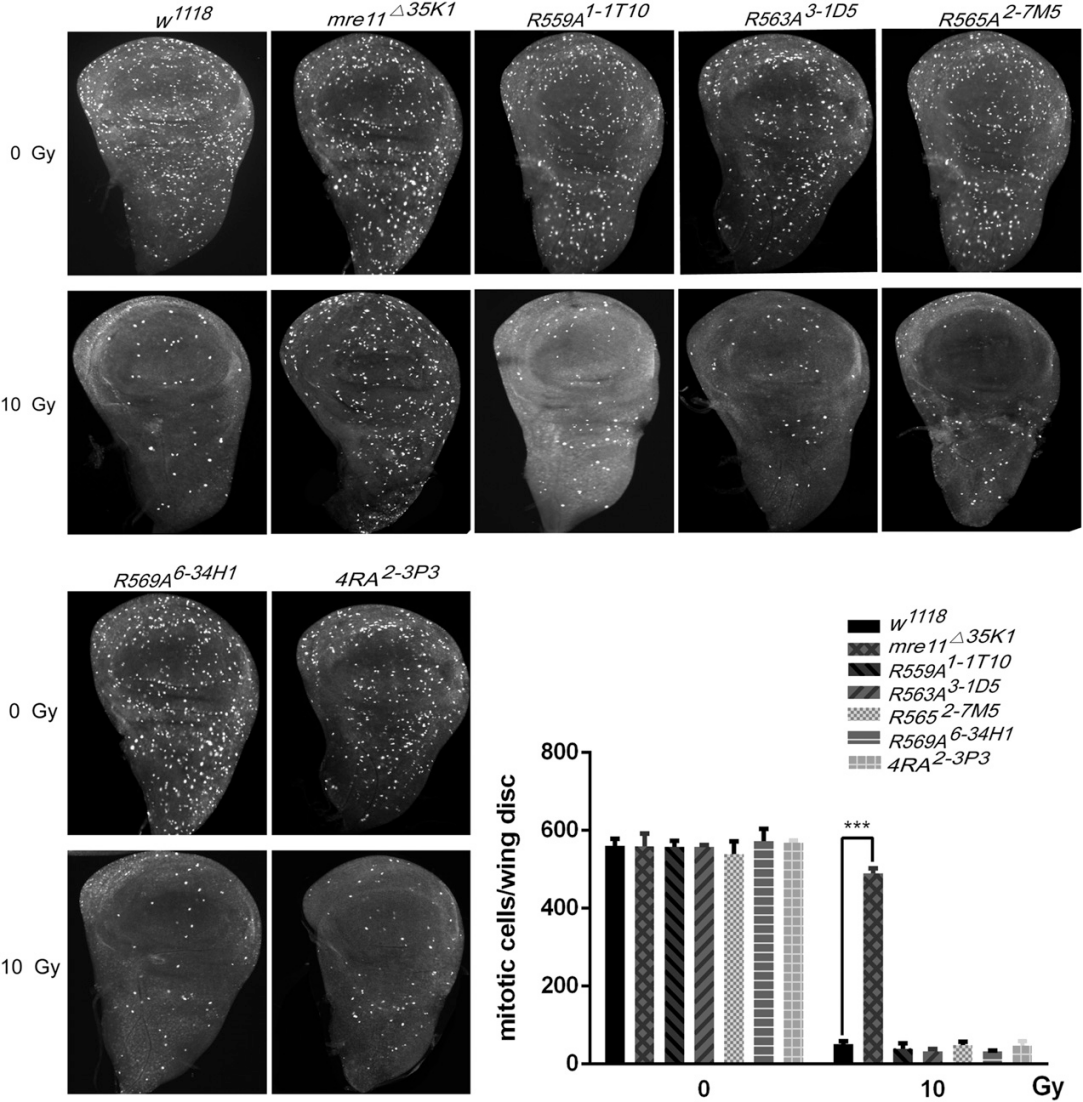
Mre11 arginines methylation is not required for irradiation induced G2/M cell cycle checkpoint. A. Photographs of wing discs of *w^1118^*, *mre11^Δ35K1^*, *mre11R559A^1-1T10^*, *mre11R563A^3-1D5^*, *mre11R565A^2-7M5^*, *mre11R569A^6-34H1^*, and *mre114RA^2-3P3^*, treated with 0 or 10 Gy, and stained with anti-histone pH 3S10 antibody. B. Quantification of mitotic cells in different genotypes. After ionizing radiation, mitosis was almost completely blocked in *w^1118^* flies, while *mre11^Δ35K1^* flies exhibited a strong G2/M checkpoint defect, and mitosis in *mre11R559A^1-1T10^*, *mre11R563A^3-1D5^*, *mre11R565A^2-7M5^*, *mre11R569A^6-34H1^*, and *mre114RA^2-3P3^* flies were almost completely blocked as in *w^1118^* flies. n ≥ 6; *** *P* ≤ 0.001; error bars indicate SEM.

We have previously shown that *mre11* knock-out flies have the DNA damage induced G2/M cell cycle checkpoint defect when treated with low-dose ionizing radiation (bi *et al.* 2005), we wish to know whether arginine methylation is required for DNA damage induced G2/M checkpoint control. We treated the third instar larvae with 10 Gy ionizing radiation as before (bi *et al.* 2005), dissected wing discs and eye discs, and stained with anti-histone H3pS10 antibody. The *mre11* knock-out flies lost the G2/M cell cycle checkpoint, while all *mre11^RA^* and *mre11^4RA^* flies had the normal G2/M cell cycle checkpoint as wild-type flies (Figure 5 and Figure S6), indicating that arginines in Mre11 GAR motif are not required for G2/M cell cycle checkpoint function in wing disc and eye disc.

## Discussion

The mammalian Mre11 is methylated at multiple arginines in its GAR motif by protein arginine methyltransferase PRMT1, and arginine methylation is critical for its exonuclease activity (boisvert *et al.* 2005a; Dery *et al.* 2008; yu *et al.* 2012), irradiation induced intra-S phase checkpoint (boisvert *et al.* 2005a) and G2/M checkpoint (yu *et al.* 2012), and Mre11 foci formation (boisvert *et al.* 2005b; Dery *et al.* 2008). As Mre11 is highly conserved in eukaryotes, whether Mre11 protein in the typical model organism *Drosophila* has arginine methylated is still an unanswered question.

In this study, we proved that the arginine methylation of Mre11 is conserved in *Drosophila*. We identified 4 arginines in the GAR motif that are methylated by DART1, the *Drosophila* homolog of PRMT1, and methylated Mre11 localized exclusively in nucleus as nucleoplasmic protein and chromatin-binding protein, suggesting the dynamic regulation of Mre11 methylation. As the GAR motif is required for Mre11 binding to DNA damage sites in mammals, and inhibition of PRMT1 activity with the pan inhibitor MTA/ADOX abrogates the Mre11-PRMT1 interaction (Dery *et al.* 2008), we investigated whether the GAR motif of *Drosophila* Mre11 is required for its interaction with DART1. Surprisingly, the *Drosophila* Mre11 lacking the GAR motif retains ability to interact with DART1, suggesting that the GAR motif is not required for their interaction in *Drosophila*. It is possible that the pan inhibitor MTA/ADOX has effects besides inhibition of Mre11 methylation, and further study is required to identify the domain that *Drosophila* Mre11 uses to interact with DART1.

Similar as knock-in *mre11^RK/RK^* mice (yu *et al.* 2012), all of the *mre11^RA^* and *mre11^4RA^* flies were viable, and showed sensitivity to ionizing radiation, which suggest that the methylation of arginines of Mre11 are not essential for viability both in mice and flies. The *mre11^RA^* and *mre11^4RA^* flies did not show irradiation induced G2/M cell cycle checkpoint defect, which is contrary to that of mammalian Mre11 functions. Therefore, the physiological significance of arginine methylation of *Drosophila* Mre11 and the underlying mechanism of its effect on sensitivity to ionizing radiation needs to be further investigated.
